# Comparative proteomics analysis of proteins expressed in the I-1 and I-2 internodes of strawberry stolons

**DOI:** 10.1186/1477-5956-9-26

**Published:** 2011-05-14

**Authors:** Xianping Fang, Huasheng Ma, Dezhao Lu, Hong Yu, Wenguo Lai, Songlin Ruan

**Affiliations:** 1 Laboratory of Plant Molecular Biology and Proteomics, Institute of Biology, Hangzhou Academy of Agricultural Sciences, Hangzhou 310024, China; 2 School of Life Science, Zhejiang Chinese Medical University, Hangzhou 310053, China

## Abstract

**Background:**

Strawberries (*Fragaria ananassa*) reproduce asexually through stolons, which have strong tendencies to form adventitious roots at their second node. Understanding how the development of the proximal (I-1) and distal (I-2) internodes of stolons differ should facilitate nursery cultivation of strawberries.

**Results:**

Herein, we compared the proteomic profiles of the strawberry stolon I-1 and I-2 internodes. Proteins extracted from the internodes were separated by two-dimensional gel electrophoresis, and 164 I-1 protein spots and 200 I-2 protein spots were examined further. Using mass spectrometry and database searches, 38 I-1 and 52 I-2 proteins were identified and categorized (8 and 10 groups, respectively) according to their cellular compartmentalization and functionality. Many of the identified proteins are enzymes necessary for carbohydrate metabolism and photosynthesis. Furthermore, identification of proteins that interact revealed that many of the I-2 proteins form a dynamic network during development. Finally, given our results, we present a mechanistic scheme for adventitious root formation of new clonal plants at the second node.

**Conclusions:**

Comparative proteomic analysis of I-1 and I-2 proteins revealed that the ubiquitin-proteasome pathway and sugar-hormone pathways might be important during adventitious root formation at the second node of new clonal plants.

## Background

**Figure 1:**
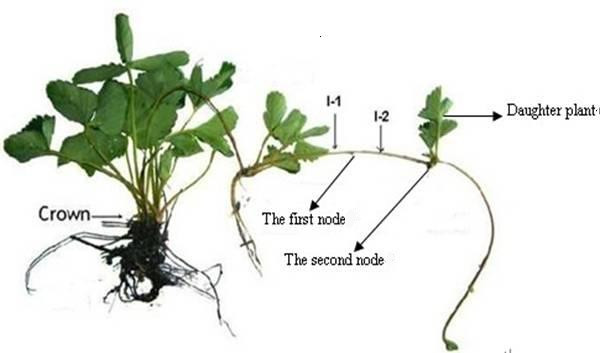
**The strawberry stolon**. As shown, a stolon shoots away from the base of a strawberry plant. A clone is formed at a variable distance away from the parent at the second node concomitant with adventitious root formation.

Several stoloniferous species produce long, sympodial stolons with rooted rosettes (ramets) at their nodes [1,2]. For the garden strawberry (*Fragaria ananassa*), the mother plant forms plantlets on stolons during spring growth (Figure 1). The first stolon originates from an auxiliary leaf bud produced in the central crown and commonly contains only two nodes. The regions along the stolon and between the plant and the first node and the first and second nodes are the I-1 (proximal) and I-2 (distal) internodes, respectively. Although stolon growth requires internode elongation, the fates of the two nodes are dissimilar. First, I-1 elongates and terminates at the first node, which is nonproductive, then I-2 elongates and terminates at the second node, which forms the main crown of the clonal plant.

Many studies have assessed the relationships between plant genotype and phenotype [3] using morphological differences caused by loss of function or altered expression of a single gene. To fully understand the function of a gene, however, the expressed protein must be characterized. Proteomics investigates the synthesis, turnover, and modification of proteins so that gene function and genotypes can be understood [4]. For example, a proteomic study of ripening strawberry fruit from plants of different genotypes identified constitutively and differentially expressed proteins that probably control the quality of the fruit [5]. Proteomics may, therefore, be used to address biochemical and physiological aspects of plant morphologies. Such approaches are increasingly used to elucidate the biochemistry and physiology of model species [6,7]. Yet proteomics is limited by the ability to identify proteins, which relies on the availability of sequence data. For plant species with unsequenced genomes, proteomics can still be applied but the number of identified proteins is usually smaller because their identification relies on homology with proteins of other species. For example, although *F. ananassa *sequence data is very limited, two proteomic techniques, namely two-dimensional gel electrophoresis (2-DE) and mass spectrometry (MS), have been used to identify an *F. ananassa *protein homologous to the birch pollen allergen Bet v 1 [8].

Elongation of the strawberry stolon is considered to be the result of cell division and cell expansion [9], but little is known about how the I-1 and I-2 internodes develop. Such knowledge is required, however, if we are to improve the cultivation of nursery strawberries and understand in greater depth how clonal multiplication occurs.

For the work reported herein, we compared the proteomes of the *F. ananassa *I-1 and I-2 internodes to elucidate the differences in their growth and functional characteristics and establish reference maps by identifying the protein spots of their 2-DE maps in conjunction with MS peptide mass determination and database searches. We identified isoforms of several proteins and present a detailed analysis of the two proteomes, which allows us to begin to explore the different developmental mechanisms of the I-1 and I-2 internodes. The reference maps should be useful for investigation of strawberry physiology and for monitoring changes in protein expression in strawberry stolons in response to biotic and abiotic stresses.

## Materials and methods

### Plant material

The *F. ananassa *cultivar Hongjia was obtained from a nursery at the Hangzhou Academy of Agricultural Sciences, Zhejiang, China. The plants were grown in a tunnel greenhouse with a 10-h light/14-h dark cycle, a 30°C-day/26°C-night temperature cycle, 150 μmol m^-2 ^s^-1 ^light intensity, and a relative humidity of 60%. Plants were watered regularly and provided adequate nutrients. In the morning, after transplantation, stolons that had formed from the leaf buds of three-month-old plants were collected and cut to isolate the I-1 and I-2 internodes (Figure 1). Daughter plants that had formed at the apices of stolons had not been allowed to root. For each experiment, 50 stolons were randomly chosen and removed from four or five plants. The I-1 and the I-2 internodes were pooled separately, rinsed with water to remove contaminants, quickly dried with paper towels, frozen in liquid nitrogen, and stored at -80°C prior to protein extraction.

### Protein extraction

Proteins were extracted using acetone and trichloroacetic acid method. A portion (2 g) of each internode sample was pulverized with a pestle in a mortar that contained liquid nitrogen and then homogenized in 10 mL of 10% (w/v) trichloroacetic acid, 0.07% (v/v) 2-sulfanylethanol in acetone. Total protein was precipitated for 1 h or overnight at -20°C. The extracts were each centrifuged at 13000 × *g *for 20 min at 4°C. The pellets were washed three times with 0.07% (v/v) 2-sulfanylethanol in acetone, and vacuum dried for 30 min. The dried powders (30 mg) were each resuspended in 500 μL of 7 M urea, 2 M thiourea, 4% 3-[(3-cholamidopropyl) dimethylammonio]-1-propanesulfonate (CHAPS), 0.75% dithiothreitol (DTT), 0.5% Biolyte (pH 3.0-10.0, Bio-Rad), 1 mM phenylmethanesulfonyl fluoride, and then shaken vigorously for 1 h at room temperature. Insoluble material was removed by centrifugation at 13000 × *g *for 15 min at 20°C. At least three replicates were prepared. Protein concentrations were determined using Bio-Rad Protein Assay kit reagents (standard Bradford method) with bovine serum albumin as the calibration standard [10].

### Two-dimensional gel electrophoresis

Each sample contained 300 μg protein in 350 μL of 8 M urea, 2 M thiourea, 2% CHAPS, 0.5% Biolyte (pH 3-10), 0.75% M DTT, 0.002% Bromophenol Blue. Each sample was each loaded onto a 17-cm immobilized pH (3-10) gradient strip (Bio-Rad). The strips were rehydrated for 12 h at 50 V. Isoelectric focusing used a linear ramp from 0 to 250 V in 15 min, a linear ramp from 250 to 10000 V in 1 h, and 10000 V for 5 h, all at 20°C. After isoelectric focusing, the strips were equilibrated in 50 mM Tris-HCl, pH 8.8, 6 M urea, 20% glycerol, 2% sodium dodecyl sulfate (SDS), 2% DTT, and then in a solution of the same composition that also contained 2.5% (w/v) iodoacetamide, (the time of each incubation was 15 min). The strips were then each placed onto a 1-mm-thick SDS (12.5% (w/v)) polyacrylamide gel and sealed with 1% (w/v) agarose. Electrophoresis was carried out in a Bio-Rad PROTEAN apparatus at 24 mA/gel. The gels were stained using a modified silver-staining method that is compatible with MS [11]. Image analysis was subsequently performed. These procedures were replicated three times.

### Image acquisition and analysis

The three replicates of the I-1 and I-2 2-DE gels were scanned using a calibrated densitometer (GS-800, Bio-Rad), and the spot patterns were characterized using PDQuest software (ver. 8.0.1, Bio-Rad). Image analysis steps included image filtration, spot detection and measurement, background subtraction, and spot matching. One I-1 gel served as the reference, and the spots of the other five gels were referenced to it. Initially, spots were automatically matched, and the positions of unmatched spots were then manually determined. The molecular mass (kDa) of each protein was estimated by comparison with those of a standard marker set, and the isoelectric points (pIs) were determined by the spot positions along the immobilized pH gradient strips.

### In-gel protein digestion and mass spectrometry

The silver-stained protein spots were manually excised from the gels, and each was placed into a well of a 96-well microplate. The gel pieces were destained in a solution prepared from a 1:1 (v/v) mixture of 30 mM potassium ferricyanide and 100 mM sodium thiosulfate at room temperature for 10 min, vortexed until destained, washed three times with 300 μL of Milli-Q water (each time for 5 min) and dehydrated in 150 μL of acetonitrile. Then the gel samples were swollen in 50 mM NH_4_HCO_3 _containing 12.5 ng/μL trypsin (Sigma, Cat. No. 089K6048) at 4°C for 30 min, and at 37°C for longer than 12 h. For each digest, the peptides were extracted from the gels twice with 5% trifluoroacetic/50% acetonitrile at room temperature, resuspended in 0.7 μL of 0.2 M α-cyano-4-hydroxy-cinnamic acid (Sigma) in 0.1% trifluoroacetic/50% acetonitrile, and allowed to dry under a stream of nitrogen. The extracted peptides were subjected to matrix-assisted laser desorption/ionization time-of-flight MS (4800 Proteomic Analyzer Applied Biosystems). Proteins were identified using the Peptide Mass Fingerprinting module of Mascot (Matrix Science) and the experimental masses of the peptides. We searched the Swissprot database in September 2010 (version 20100906, which included 519348 sequences and 183273162 residues) for proteins from *Viridiplantae *(green plants, 29439 sequences). One missed cleavage per peptide was allowed, and a mass tolerance of 50-150 ppm was used. Carbamidomethylation of cysteine was set as a fixed modification, and oxidation of methionine was allowed. Identified proteins with a peptide mass fingerprint were denoted as having an unambiguous identification by the following criteria: (1) at least five different predicted peptide masses were needed to match the observed masses for an identification to be considered valid; and (2) protein scores needed to have >57 identity for Swissprot database (p < 0.05).

### Gene ontology (GO) annotation and protein classification

The UniProt database (http://www.uniprot.org) was searched to determine the functions of the identified proteins. Three independent ontological sets in the *Viridiplantae *taxonomic database were used to annotate and group the proteins according to biological processes, molecular function, and cellular compartmentalization.

### Protein-protein interactions (PPIs)

PPIs were predicted by Cytoprophet, which is a plug-in of Cytoscape. We sent all the data for the identified proteins in I-1 and I-2 to the Cytoprophet server along with the set cover approach maximum specificity set cover (MSSC). The algorithm was used to predict the interaction network(s).

## Results and discussion

### Proteome analysis and protein identification

**Figure 2:**
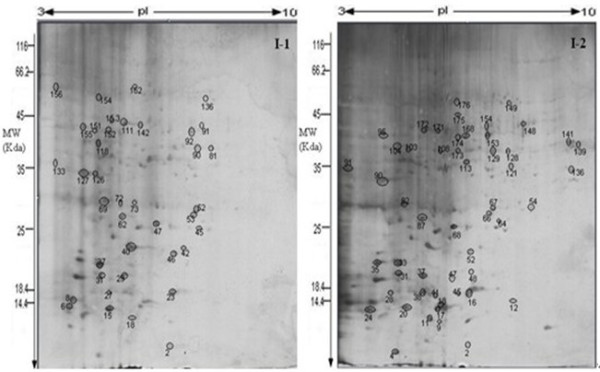
**Two-dimensional SDS-PAGE gels of the I-1 and I-2 proteomes**. Proteins (300 μg) in I-1 and I-2 extracts were separated, in the first dimension by isoelectric focusing (pH 3-10) and in the second dimension by SDS-PAGE through 12.5% acrylamide gels. Proteins were visualized by silver staining. Circled proteins were identified by matrix-assisted laser desorption/ionization time-of-flight MS and database searches.

We investigated the differences between the I-1 and 1-2 protein profiles. More than 503 I-1 and 1127 I-2 protein spots were reproducibly detected (Figure 2). The pIs of the protein spots ranged from 3.5 to 9.3, and the molecular masses ranged from 7.1 to 60.2 kDa.

**Figure 3:**
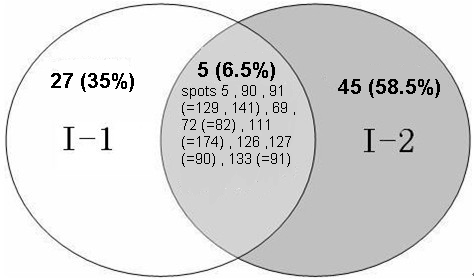
**Venn diagram for the identified I-1 and I-2 proteins**. The numbers and percentages of unique proteins (excluding isoforms) found for either or both internodes are given. Spot numbers for I-1 proteins are given first followed by spot numbers for the corresponding I-2 proteins in parentheses.

**Figure 4:**
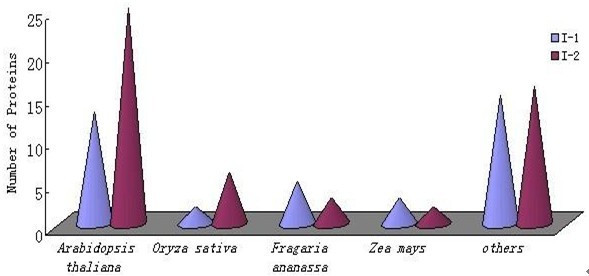
**Number of proteins with sequences that matched those of organisms listed in the Swissprot *Viridiplantae *database**. Over 50% of the proteins identified had sequences similar to annotated proteins from dicot species in the *Viridiplantae *database.

For protein identification, the peptide mass fingerprinting data were used in conjunction with a search of the complete Swissprot *Viridiplantae *taxonomic database as only 1017 *F. ananassa *protein sequences were available therein. Respectively, 164 and 200 spots from the I-1 and I-2 gels were selected for MS, and 38 and 52 proteins were identified, i.e., ~25% of the total in each case (Tables 1 and 2). No unambiguous matches were made for the other 274 proteins, probably because the proteins were not included in the database or because a protein spot contained more than one protein. For the 90 identified proteins, 5 (6.5%) were common to both proteomes, which indicated that the two internodes had some proteins (probably housekeeping proteins) in common. Certain proteins (35% of the gene products) were specific to I-1, whereas 58.5% were specific to I-2 (Figure 3). More than 50% of the proteins could be correlated with annotated proteins from at least one dicotyledon species (Figure 4). Of the *F. ananassa *proteins identified in our study, 13 I-1 and 25 I-2 proteins were matched to *Arabidopsis thaliana *proteins, and others were matched to *Oryza sativa *proteins (two from I-1, six from I-2) and *Zea mays *proteins (three from I-1, two from I-2). Only five I-l proteins and three I-2 proteins could be matched to those found in the *F. ananassa *database, possibly because the number of annotated proteins contained in the database is relatively small compared with that for the *A. thaliana *database.

**Table 1:** Identified proteins from the strawberry stolon I-1 internode

**Spot No.**^***a***^	Protein	**Accession Number**^***b***^	**Molecular Function**^***c***^	Reference Organism	**Theoretical kDa/pI**^***d***^	**Experimental kDa/pI**^***e***^	**Score**^***f***^	**SC**^***g ***^**(%)**	**Matched/Unmatched queries**^***h***^
	**Metabolism**								
15	Malate dehydrogenase	P83373	L-malate dehydrogenase activity	*Fragaria ananassa*	35.8/8.7	13.5/5.3	99	41	9/43
29	Glucan endo-1,3-beta-glucosidase 6	Q93Z08	glucan endo-1,3-beta-D-glucosidase activity	*Arabidopsis thaliana*	52.6/5.6	20.3/5.8	58	27	6/35
31	Glucan endo-1,3-beta-glucosidase 6	Q93Z08	glucan endo-1,3-beta-D-glucosidase activity	*Arabidopsis thaliana*	52.6/5.6	20.4/5.0	63	21	5/42
40	Uricase	O04420	urate oxidase activity	*Arabidopsis thaliana*	35.0/8.6	23.4/6.1	58	38	10/107
42	GDSL esterase/lipase At4g16220	O23469	hydrolase activity, acting on ester bonds	*Arabidopsis thaliana*	26.7/8.9	23.2/7.8	57	37	6/36
90	Malate dehydrogenase, mitochondrial	P83373	L-malate dehydrogenase activity	*Fragaria ananassa*	35.8/8.7	40.3/8.2	122	51	10/50
91	Malate dehydrogenase, mitochondrial	P83373	L-malate dehydrogenase activity	*Fragaria ananassa*	35.8/8.7	43.3/8.3	100	50	9/45
111	S-adenosylmethionine synthase	Q8W3Y4	metal ion binding	*Phaseolus lunatus*	43.5/5.6	47.4/5.8	74	24	7/9
118	2,3-bisphosphoglycerate-independent phosphoglycerate mutase	P30792	manganese ion binding	*Zea mays*	60.7/5.2	41.3/4.9	59	24	8/27
133	Fructokinase-1	A2WXV8	ATP binding	*Oryza sativa*	34878/5.1	36.1/3.8	76	23	6/15
142	S-adenosylmethionine synthase 4	A9PHC5	metal ion binding	*Populus trichocarpa*	42999/5.7	46.5/6.3	74	18	5/2
162	Phosphoglucomutase	P93804	magnesium ion binding	*Zea mays*	63286/5.4	60.2/6.1	63	17	9/30
	**Energy**								
152	ATP synthase subunit beta, chloroplastic	Q9MRR9	ATP binding	*Brasenia schreberi*	53.8/5.2	45.3/4.8	76	27	8/19
153	ATP synthase subunit beta	Q01859	ATP binding	*Oryza sativa*	59.0/5.9	48.1/5.4	134	44	19/45
155	ATP synthase subunit beta	Q6QBP2	ATP binding	*Castanea sativa*	53.8/5.3	45.9/4.4	108	37	15/26
156	ATP synthase subunit alpha	A4QJA0	ATP binding	*Aethionema cordifolium*	55.3/5.2	58.5/3.5	114	22	10/13
	**Photosynthesis**								
2	Ribulose bisphosphate carboxylase small chain 1A	P10795	monooxygenase activity	*Arabidopsis thaliana*	20.5/7.6	10.7/7.8	62	43	7/101
37	Ribulose bisphosphate carboxylase large chain (Fragment)	O98681	ribulose-bisphosphate carboxylase activity	*Zamioculcas zamiifolia*	49.9/6.3	21.4/4.9	79	25	10/78
46	Ribulose bisphosphate carboxylase large chain	P28439	magnesium ion binding	*Pelargonium hortorum*	53.3/6.3	23.7/7.5	62	32	13/104
69	Triosephosphate isomerase	Q9M4S8	triose-phosphate isomerase activity	*Fragaria ananassa*	33.7/7.6	30.0/5.1	152	55	19/112
72	Triosephosphate isomerase	Q9M4S8	triose-phosphate isomerase activity	*Fragaria ananassa*	33.7/7.6	31.3/5.6	126	59	16/77
126	Oxygen-evolving enhancer protein 1	P26320	calcium ion binding	*Solanum tuberosum*	35.6/5.8	35.6/4.4	111	33	8/8
127	Oxygen-evolving enhancer protein 1	P26320	calcium ion binding	*Solanum tuberosum*	35.6/5.8	34.9/4.8	113	28	9/21
	**Transcription**								
6	Histone acetyltransferase GCN5	Q9AR19	protein binding	*Arabidopsis thaliana*	63.5/6.0	14.2/4.1	59	29	11/66
18	Pentatricopeptide repeat-containing protein At3g09650	Q9SF38	nucleotide binding	*Arabidopsis thaliana*	84.4/6.5	11.2/6.2	80	23	14/49
27	Transcription factor bHLH145	Q9FGB0	DNA binding	*Arabidopsis thaliana*	35.2/5.1	18.3/5.1	62	28	10/63
92	DNA-directed RNA polymerase subunit beta	B1VKH5	DNA-directed RNA polymerase activity	*Cryptomeria japonica*	139.6/9.4	44.6/8.1	59	12	11/65
136	DNA polymerase	P10582	3'-5' exonuclease activity	*Zea mays*	108.2/8.6	55.3/8.5	58	15	8/23
	**Protein synthesis**								
154	50S ribosomal protein L33	B2LML4	structural constituent of ribosome	*Guizotia abyssinica*	7.8/9.7	56.4/5.1	62	42	5/11
	**Protein folding, degradation and assembly**								
45	Heat shock protein 81-3	P51818	ATP binding	*Arabidopsis thaliana*	80.2/5.0	26.7/8.2	59	19	9/85
73	Proteasome subunit alpha type-6	O48551	threonine-type endopeptidase activity	*Glycine max*	27.5/5.8	28.3/6.1	58	31	6/62
	**Transport**								
23	NAD(P)H-quinone oxidoreductase subunit H, chloroplastic OS	A6MMH1	oxidoreductase activity, acting on NADH or NADPH	*Chloranthus spicatus*	45.8/5.4	18.5/7.3	75	26	12/66
52	Ras-related protein ARA-4	P28187	GTP binding	*Arabidopsis thaliana*	24.1/5.0	28.6/8.4	61	28	10/101
	**Stress Related**								
62	Putative F-box/kelch-repeat protein At4g19330	O65704	N/A	*Arabidopsis thaliana*	62.9/7.1	27.3/5.7	59	25	10/82
151	Putative F-box/kelch-repeat protein At4g19330	O65704	N/A	*Arabidopsis thaliana*	62.8/7.1	44.3/4.8	65	21	8/75
	**Development**								
53	3-ketoacyl-CoA synthase 5	Q9C6L5	fatty acid elongase activity	*Arabidopsis thaliana*	56.3/9.0	27.7/8.1	61	40	12/19
81	Cytoplasmic dynein 2 heavy chain 1	Q9SMH5	ATP binding	*Chlamydomonas reinhardtii*	483.5/6.1	40.0/8.6	58	7	22/48
	**Unknown**								
8	Embryonic abundant protein VF30.1 OS = Vicia faba PE = 2 SV = 1	P21745	N/A	*Vicia faba*	30.1/6.4	18.1/4.2	67	33	7/67

^*a*^As indicated in Figure 2. ^*b*^Swissprot database accession number. ^*c*^Molecular functions were inferred from those reported in the UniProt database. ^*d*^Theoretical isoelectric point and molecular mass. ^*e*^Experimental isoelectric point and molecular mass. ^*f*^Mascot score. ^*g*^Sequence coverage. ^*h*^Number of matched and unmatched peptides.

**Table 2:** Identified proteins from the strawberry stolon I-2 internode

**Spot No.**^***a***^	Protein	**Accession Number**^***b***^	**Molecular Function**^***c***^	Reference Organism	**Theoretical kDa/pI**^***d***^	**Experimental kDa/pI**^***e***^	**Score**^***f***^	**SC**^***g ***^**(%)**	**Matched/Unmatched queries**^***h***^
	**Metabolism**								
91	Fructokinase-1	A2WXV8	ATP binding	*Oryza sativa*	34.9/5.1	35.8/3.4	76	20	6/59
95	Caffeic acid 3-O-methyltransferase	Q8GU25	caffeate O-methyltransferase activity	*Rosa chinensis*	40.1/5.6	43.7/4.4	110	35	12/30
129	Malate dehydrogenase, mitochondrial	P83373	Oxidoreductase	*Fragaria ananassa*	35.8/8.7	39.1/7.8	74	31	8/28
139	Glyceraldehyde-3-phosphate dehydrogenase, cytosolic	P25858	Oxidoreductase	*Arabidopsis thaliana*	37.0/6.6	40.1/9.3	74	29	9/27
141	Malate dehydrogenase, mitochondrial	P83373	Oxidoreductase	*Fragaria ananassa*	35.8/8.7	41.0/9.1	65	27	6/23
172	S-adenosylmethionine synthase 2	Q9FUZ1	ATP binding	*Brassica juncea*	43.3/5.3	43.9/5.9	138	44	15/36
173	S-adenosylmethionine synthase	A4PU48	ATP binding	*Medicago truncatula*	43.7/5.6	38.5/6.8	178	52	15/17
174	S-adenosylmethionine synthase	Q8W3Y4	ATP binding	*Phaseolus lunatus*	43.5/5.6	41.2/6.7	132	48	15/40
	**Energy**								
176	ATP synthase subunit beta, plastid	Q8MBG5	ATP binding	*Cuscuta pentagona*	53.2/5.5	53.4/6.7	61	32	10/53
	**Photosynthesis**								
17	Probable alpha,alpha-trehalose-phosphate synthase [UDP-forming] 4OS	Q9T079	alpha,alpha-trehalose-phosphate synthase (UDP-forming) activity	*Arabidopsis thaliana*	90.3/6.1	14.2/6.3	58	25	12/66
33	Ribulose bisphosphate carboxylase large chain (Fragment) OS	P28261	magnesium ion binding	*Nypa fruticans*	51.5/6.2	22.3/5.1	123	23	13/61
35	Probable granule-bound starch synthase 1, chloroplastic/amyloplastic	Q9MAQ0	starch synthase activity	*Arabidopsis thaliana*	67.5/8.7	23.0/4.5	60	24	9/37
52	Ribulose bisphosphate carboxylase large chain (Fragment)	P28391	magnesium ion binding	*Ceratopetalum gummiferum*	51.3/6.2	24.0/6.9	104	22	11/63
64	Ribulose-phosphate 3-epimerase, chloroplastic	Q43157	ribulose-phosphate 3-epimerase activity	*Spinacia oleracea*	30.6/8.2	27.2/7.9	58	31	6/51
82	Triosephosphate isomerase, chloroplastic	Q9M4S8	triose-phosphate isomerase activity	*Fragaria ananassa*	33.7/7.6	31.5/5.2	154	51	16/71
90	Oxygen-evolving enhancer protein 1, chloroplastic	P26320	calcium ion binding	*Solanum tuberosum*	35.6/5.8	34.5/4.7	98	31	10/48
103	Coproporphyrinogen-III oxidase, chloroplastic	P35055	coproporphyrinogen oxidase activity	*Glycine max*	43.6/6.7	42.2/5.2	84	31	10/32
104	Glutamyl-tRNA reductase 1, chloroplastic	Q42843	NADP or NADPH binding	*Hordeum vulgare*	58.1/8.7	42.1/4.9	70	24	11/48
	**Transcription**								
18	Homeobox-leucine zipper protein GLABRA 2 OS	P46607	sequence-specific DNA binding	*Arabidopsis thaliana*	83.7/6.1	15.0/6.4	63	31	20/90
47	Protein HIRA	Q32SG6	transcription regulator activity	*Zea mays*	106.55/7.8	19.7/7.1	60	22	16/90
121	Two-component response regulator ARR9	O80366	two-component response regulator activity	*Arabidopsis thaliana*	26.2/5.2	34.6/8.1	58	35	5/43
128	DNA-directed RNA polymerase subunit beta	P12465	DNA binding	*Chlorella vulgaris*	179.4/9.9	36.9/8.0	60	9	11/34
149	B3 domain-containing protein Os07g0679700	Q6Z3U3	DNA binding	*Oryza sativa*	105.4/6.5	53.5/7.9	63	11	9/27
168	Protein HIRA	Q32SG6	transcription regulator activity	*Zea mays*	106.5/7.8	41.1/6.8	60	22	16/90
	**Protein synthesis**								
28	Eukaryotic translation initiation factor 5A-2 OS	Q945F4	translation initiation factor activity	*Medicago sativa*	17.5/5.4	17.3/4.7	74	26	5/39
37	Eukaryotic translation initiation factor 5A	Q9AXQ7	translation initiation factor activity	*Dianthus caryophyllus*	17.6/5.6	20.3/5.9	88	45	7/28
45	Glutathione gamma-glutamylcysteinyltransferase 2	Q9ZWB7	acyltransferase activity	*Arabidopsis thaliana*	52.3/6.6	16.4/6.6	57	15	8/32
66	Phospho-2-dehydro-3-deoxyheptonate aldolase 1, chloroplastic	P21357	3-deoxy-7-phosphoheptulonate synthase activity	*Solanum tuberosum*	60.0/8.9	27.9/7.3	62	31	11/71
87	Molybdenum cofactor sulfurase	Q655R6	lyase activity	*Oryza sativa*	92.9/7.1	27.2/5.2	60	12	6/22
136	Probable beta-1,3-galactosyltransferase 18	Q8RX55	galactosyltransferase activity	*Arabidopsis thaliana*	77.9/8.7	35.1/9.1	66	17	7/27
	**Protein folding, degradation and assembly**								
148	Anaphase-promoting complex subunit 2	Q8H1U5	ubiquitin protein ligase binding	*Arabidopsis thaliana*	98.4/4.8	43.5/7.9	64	12	7/20
153	U-box domain-containing protein 34	Q8S8S7	ubiquitin-protein ligase activity	*Arabidopsis thaliana*	91.7/9.1	39.6/6.9	63	18	12/51
171	Ubiquitin carboxyl-terminal hydrolase 6	Q949Y0	ubiquitin-specific protease activity	*Arabidopsis thaliana*	54.0/5.8	41.6/5.8	58	19	8/35
175	U-box domain-containing protein 34	Q8S8S7	ubiquitin-protein ligase activity	*Arabidopsis thaliana*	91.7/9.1	44.7/6.2	58	19	12/67
	**Transport**								
24	Magnesium transporter MRS2-8 OS	Q8H1G7	metal ion transmembrane transporter activity	*Arabidopsis thaliana*	43.1/5.3	13.6/4.4	58	22	6/34
54	Putative copper-transporting ATPase 3	Q9SH30	ATP binding	*Arabidopsis thaliana*	109.0/6.0	29.0/8.0	65	18	14/48
	**Stress Related**								
4	Ninja-family protein AFP4 OS	Q9S7Z2	protein binding	*Arabidopsis thaliana*	35.6/8.5	7.1/5.0	57	40	9/70
9	Glutathione S-transferase 6	Q96266	glutathione binding	*Arabidopsis thaliana*	29.2/8.5	11.9/6.2	58	25	5/46
20	Annexin D6 OS	Q9LX08	calcium ion binding	*Arabidopsis thaliana*	36.6/7.7	14.0/5.1	62	20	7/39
41	Monodehydroascorbate reductase, chloroplastic	P92947	monodehydroascorbate reductase (NADH) activity	*Arabidopsis thaliana*	53.5/8.1	16.2/5.7	63	26	8/76
154	Monodehydroascorbate reductase	Q40977	monodehydroascorbate reductase (NADH) activity	*Pisum sativum*	47.3/5.8	43.5/6.8	58	22	7/31
	**Development**								
2	Pentatricopeptide repeat-containing protein At3g06430, chloroplastic OS	Q9SQU6	N/A	*Arabidopsis thaliana*	56.2/7.8	9.5/6.7	58	25	11/59
11	Protein BRUSHY 1	Q6Q4D0	protein binding	*Arabidopsis thaliana*	148.8/5.5	12.0/5.9	63	24	21/71
38	Protein PAIR1	Q75RY2		*Oryza sativa*	53.8/9.8	17.2/5.5	66	19	10/34
68	1-aminocyclopropane-1-carboxylate synthase 7	Q9STR4	1-aminocyclopropane-1-carboxylate synthase activity	*Arabidopsis thaliana*	51.0/5.9	26.2/6.3	57	19	10/72
	**Unknown**								
12	Probable protein ABIL5 OS	Q5JMF2	N/A	*Oryza sativa*	28.3/8.3	15.2/7.6	59	40	7/42
16	Pentatricopeptide repeat-containing protein At4g26800 OS	Q9SZ20	N/A	*Arabidopsis thaliana*	58.4/9.1	16.1/6.8	64	32	12/49
31	Putative F-box protein At1g20795 OS	Q9LM74	N/A	*Arabidopsis thaliana*	48.5/8.6	20.3/5.1	60	21	8/55
48	Probable protein ABIL5 OS	Q5JMF2	N/A	*Oryza sativa*	28.2/8.2	21.5/6.9	59	40	7/42
67	BRCT domain-containing protein At4g02110	O04251	N/A	*Arabidopsis thaliana*	142.5/8.4	28.4/7.5	59	19	18/80
108	Thylakoid lumenal 15.0 kDa protein 2, chloroplastic	Q9LVV5	N/A	*Arabidopsis thaliana*	24.7/5.7	37.8/5.8	68	26	7/37
113	Pentatricopeptide repeat-containing protein At2g01860	Q5XET4	N/A	*Arabidopsis thaliana*	56.0/9.2	36.1/6.8	59	39	15/95

^*a*^Spot number as in Figure 2. ^*b*^Swissprot database accession number. ^*c*^Molecular functions were inferred from those reported in the UniProt database. ^*d*^Theoretical molecular mass and isoelectric point. ^*e*^Experimental molecular mass and isoelectric point. ^*f*^Mascot score. ^*g*^Sequence coverage. ^*h*^Number of matched and unmatched peptides.

### Protein isoforms and subunits of protein complexes

Isoforms for malate dehydrogenase (spots 15, 90, and 91), glucan endo-1,3-β-glucosidase 6 (spots 29 and 31), triosephosphate isomerase (spots 69 and 72), oxygen-evolving enhancer protein 1 (spots 126 and 127), and a putative F-box/kelch-repeat protein (spots 62 and 151) were found in the I-1 proteome, and isoforms for malate dehydrogenase (spots 129 and 141), U-box domain-containing protein 34 (spots 153 and 175), and the probable protein ABIL5 (spots 12 and 48) were found for I-2 (Tables 1 and 2). Therefore, respectively, ~29% and ~12% of the identified I-1 and I-2 proteins exist as isoforms. Previous studies reported that ~70% of maize or *Arabidopsis *proteins exist as isoforms [12,13]. The experimental masses and/or pIs of certain proteins differed from their theoretical values, possibly owing to co-translational and/or post-translational modification (e.g., glycosylation, phosphorylation, and/or proteolysis), translation from alternatively spliced mRNAs [14-17], or post-translational modification by a nonprotein component(s) [18].

Of the 90 identified proteins, 16 (more than 17%) form complexes [19]. For example, two I-1 spots corresponded to the α and β subunits of chloroplastic ATP synthase. The β-subunit of the DNA-directed RNA polymerase subunit was detected in both the I-1 and I-2 proteomes. For some complexes, all component subunits were detected; for instance, both the small and large subunits of ribulose-bisphosphate carboxylase/oxygenase (RuBisCo) were identified in the I-1 proteome. The fact that several complexes were identified will facilitate future studies of how complex formation is regulated. The components of a protein complex would be expected to be coordinately expressed and regulated to keep a system in functional balance. Thus, our 2-DE reference maps can be used to compare the levels of functionally related polypeptides (isoforms and subunits of complexes) and may provide insights into protein function and participation in molecular networks.

### Functional classification and subcellular localization of the identified proteins

**Figure 5:**
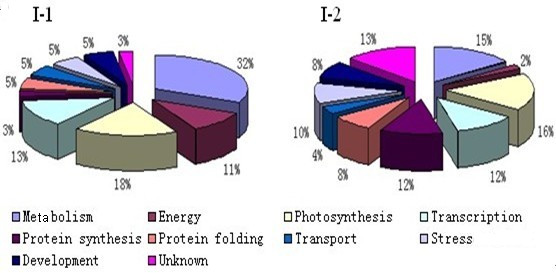
**Pie charts classifying the identified I-1 and I-2 proteins according to biological function**. The identified proteins were grouped according to their biological processes and are expressed in percentage.

**Figure 6:**
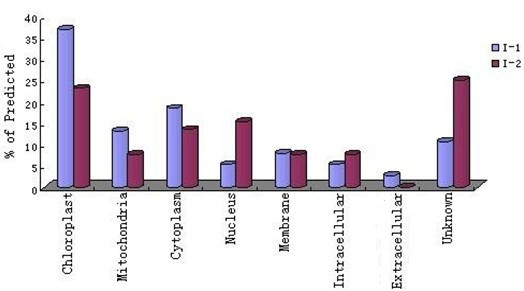
**Gene ontology classification of identified I-1 and I-2 proteins according to their subcellular location**. Subcellular locations of the proteins were assigned according to the GO annotations and are expressed as percentages of the assigned proteins.

Identification of proteins that are differentially expressed in I-1 and I-2 is important for our understanding of internodal development and differentiation. The identified proteins were grouped according to their biological processes, i.e., photosynthesis, protein synthesis, protein folding, transcription, transport, stress, and development, and cellular locations using the GO annotation in the *Viridiplantae *taxonomic databases (Figures 5 and 6). Proteins involved in metabolism and photosynthesis accounted for 32% and 18%, respectively, of the I-1 proteins. The numbers of I-1 proteins involved in transcription (13%) and energy production (8%) were also substantial. Interestingly, for I-2, although still the largest two groups, metabolic proteins accounted for only 15% and those involved in photosynthesis for 16%. I-2 proteins involved in protein synthesis and DNA transcription each accounted for 12% of the total. The function could not be determined for 3% of the identified I-1 proteins and ~13% of the I-2 proteins. The percentage of unidentified I-2 proteins is similar to those found for maize [20], rice [21] or oilseed rape [22]. The substantial differences in the numbers of I-1 and I-2 proteins involved in metabolism, energy production, and protein synthesis suggest that the functions of these proteins during development deserve further attention.

Subcellular localization provides important information about a protein's physiological function [23,24]. Recently, GO annotation has been widely used to predict the locations of proteins [25-29], because the two are strongly correlated. We found most of the identified proteins to be located in chloroplasts and mitochondria, which is congruent with total genomic data available for plants [30]. Interestingly, most of these proteins are involved in metabolism or energy production, which is consistent with the large number of proteins that we classified as metabolic or photosynthetic. We could not identify the cellular location of ~11% and ~25% I-1 and I-2 proteins, respectively.

### Proteins involved in metabolism

For I-1, identified proteins were found for lipid metabolism (spot 42), purine metabolism (spot 40), the citric acid cycle (spots 15, 90, and 91), starch and sucrose metabolism (spots 29, 31, 118, 133, and 162), and one-carbon metabolism (spots 111 and 142). For I-2, we found additional proteins involved in one-carbon metabolism (spots 172, 173, and 174) but fewer and different proteins involved in starch and sucrose metabolism (spots 91 and 139). Many more proteins involved in metabolism were found for I-1 than for I-2 (Tables 1 and 2, Figure 5). The activities of extracellular lipase (spot 42) and β-1,3-glucanase (spots 29 and 31) have been implicated in pollen germination, fertilization, response to wounding, and cell division [31,32], all or any of which may be related to I-1 elongation via cell division. Sugars are involved in energy metabolism and act as signaling molecules. For I-1, we identified 2,3-bisphosphoglycerate-independent phosphoglycerate mutase (spot 118) and phosphoglucomutase (spot 162), and for I-2, glyceraldehyde-3-phosphate dehydrogenase (spot 159), all of which are glycolytic enzymes. Interestingly, malate dehydrogenase (spots 90 and 129), fructokinase-1 (spots 155 and 91), and S-adenosyl-l-methionine (AdoMet) synthase (spots 111 and 175) were identified in both I-1 and I-2 proteomes. Malate dehydrogenase is a citric-acid-cycle enzyme that converts malate into oxaloacetate (using NAD). Fructokinase is involved in sucrose and fructose metabolism and may regulate starch synthesis in conjunction with sucrose synthase, which first metabolizes plant sink tissue in, for example, potatoes [33]. AdoMet synthase catalyzes the formation of AdoMet from methionine and ATP [34], which is the main methyl group donor and is involved in transmethylations and the trans-sulfuration pathway [35]. AdoMet is also involved in the biosynthesis of many secondary metabolites [36,37] and can be decarboxylated to generate a propylamine donor for polyamine biosynthesis [38]. Polyamines are required for cell proliferation and may play a role in the rapid growth of bloom-forming dinoflagellates [39]. In plants, AdoMet participates in ethylene biosynthesis [40] and is the methyl group donor in transmethylation of alkaloids [41]. Cell and life cycle variation in AdoMet synthase expression has been observed in yeast and apicomplexa [42,43].

### Proteins involved in energy production

More identified I-1 proteins (11%) were found to be involved in energy production than were I-2 proteins (2%, Figure 5). Both the α and β subunits of chloroplastic ATP synthase (spots 156 and 152) were identified for I-1, whereas only the ATP synthase β-subunit was detected in the I-2 proteome. ATP synthase is a very large complex (>500 kDa) embedded in the inner membranes of chloroplasts and mitochondria. It utilizes the products of fat and carbohydrate breakdown to generate proton gradients across membranes, which then drive ATP synthesis.

### Proteins involved in photosynthesis

Photosynthesis uses light energy and chlorophyll to synthesize simple sugars from carbon dioxide and water and to capture the energy as phosphate bonds in ATP. ATP is then available as an energy source, and the sugars are used as building blocks to produce other cell structural and storage components. Photosynthetic enzymes including RuBisCo (spots 2, 37, 46, and 33), oxygen-evolving enhancer protein 1 (spots 126 and 90), and triosephosphate isomerase (spots 69, 72, and 82) were found in the I-1 and I-2 proteomes.

Interestingly, many other proteins involved in sugar synthesis were found only in the I-2 proteome, i.e., alpha, alpha-trehalose-phosphate synthase (UDP-forming, spot 17), granule-bound starch synthase 1 (spot 35), and ribulose-phosphate 3-epimerase (spot 64). Alpha, alpha-trehalose-phosphate synthase (UDP-forming) synthesizes alpha, alpha-trehalose 6-phosphate from D-glucose 6-phosphate, and is then dephosphorylated to trehalose by trehalose 6-phosphate phosphatase. Trehalose metabolism, a side-branch of carbon flux in bacteria, yeast, and plants, has recently drawn attention because it may partially regulate plant growth, development, and stress resistance [44]. Granule-bound starch synthase 1 is required for the synthesis of amylase, and ribulose-phosphate 3-epimerase (pentose-5-phosphate 3-epimerase) converts d-ribulose 5-phosphate into d-xylulose 5-phosphate as part of the reductive pentose phosphate (Calvin) cycle. These aforementioned enzymes are very important for sugar synthesis. Sugars can act as signaling molecules in microorganisms, animals, and plants. During plant growth and development, sugars modulate seed germination, seedling development, root and leaf differentiation, floral transition, fruit ripening, embryogenesis, senescence, and responses to light, stress, and pathogens [45-53]. For strawberries, the clonal plant is usually found at the second node, so the identification of specific sugar-related enzymes found only in the I-2 proteome should increase our understanding of the different internodal developmental mechanisms. Our results indicate that positive interactions between sugar synthesis and hormonal signaling in I-2 may be necessary for asexual strawberry reproduction.

### Proteins involved in protein synthesis

We identified only one I-1 protein associated with protein synthesis, namely the 50S ribosomal protein L33 (spot 154). Cell growth and division require the synthesis of new proteins and ribosomes. L33 is involved in the biogenesis of both the small and large ribosomal subunits. For I-2, we found the eukaryotic translation initiation factor 5A (spots 28 and 37), which is a highly conserved eukaryotic protein. Eukaryotic translation initiation factor 5A appears to be involved in RNA metabolism and trafficking in mammals and yeast, thus regulating cell proliferation, cell growth, and programmed cell death [54]. In plants, however, its physiological function is not known.

### Proteins involved in protein folding and protein degradation

For I-1, two proteins involved in protein folding and processing were identified, and for I-2, four proteins involved in protein modification and degradation were found. The heat shock protein 81-3 (spot 45) found in the I-1 proteome is a molecular chaperone and likely involved in signal transduction and development associated with certain hormone receptors and kinases [55]. Interestingly, three of the I-2 proteins, anaphase-promoting complex subunit 2 (APC2; spot 148), U-box domain-containing protein 34 (spots 153 and 175), and ubiquitin carboxyl-terminal hydrolase 6 (spot 171), are all ubiquitin-dependent proteins involved in catabolism. The ubiquitin-proteasome pathway is responsible in large part for protein degradation and consequently regulates many aspects of development. The identification of the aforementioned proteins suggests that research on the ubiquitin conjugation pathway might illuminate the mechanism of I-2 clonal multiplication.

### Proteins involved in transcription

We identified five I-1 proteins, histone acetyltransferase GCN5 (spot 6), transcription factor bHLH145 (spot 27), DNA polymerase (spot 136), pentatricopeptide repeat-containing protein (spot 18), and DNA-directed RNA polymerase (spot 92), that are involved in transcription. GCN5 is a coactivator of transcriptional regulation [56,57]. The I-2 homeodomain-leucine zipper protein (spot 18) is a putative transcription factor required for correct morphological development and maturation of trichomes as well as for normal development of seed coat mucilage [58]. The function of histone regulator protein (spots 47 and 168) has yet to be determined; however, it may be involved in maintining knox genes silencing throughout leaf development [59]. The two-component response regulator (spot 121) is involved in the His-to-Asp phosphorelay signal transduction system [60].

### Proteins involved in stress response and development

Twelve of the identified I-1 and I-2 proteins are associated with stress and development. One I-1 protein (spots 62 and 151) and five I-2 proteins (e.g., spots 4, 9, 20, 41, and 154) are involved in stress responses, and two I-1 proteins (spots 53 and 81) and four I-2 proteins (spots 2, 11, 38, and 68) are associated with development. Spot 53 is 3-ketoacyl-CoA synthase, which mediates the synthesis of very long chain fatty acids (26 to 30 carbons). Spot 81 is the cytoplasmic dynein 2 heavy chain1, which is an intracellular motor for retrograde vesicle and organelle motility along microtubules.

For I-2, monodehydroascorbate reductase (spots 41 and 154) was identified and is an oxidoreductase that oxidizes NADH or NADPH using a quinone as the oxidant during the glutathione-ascorbate cycle, a major plant antioxidant system that protects against reactive oxygen species. Monodehydroascorbate reductase activity has been found in chloroplasts, the cytosol, mitochondria, glyoxysomes, and leaf peroxisomes [61]. 1-aminocyclopropane-1-carboxylate synthase 7 (spot 68) catalyzes the conversion of AdoMet into 1-aminocyclopropane-1-carboxylate, a precursor of ethylene.

### Comparison of metabolic pathways in I-1 and I-2

**Figure 7:**
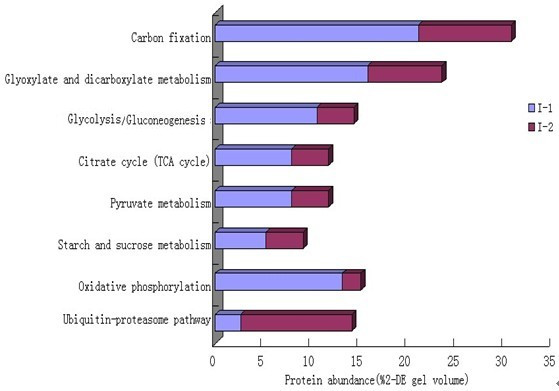
**Protein abundance for nine metabolic pathways in I-1 and I-2**. For carbon fixation, glyoxylate and dicarboxylate metabolism, glycolysis/gluconeogenesis, pentose phosphate pathway, pyruvate metabolism, starch and sucrose metabolism, oxidative phosphorylation, ubiquitin-proteasome pathway; 7, 6, 4, 1, 1, 3, 6, and 5 enzymes were identified, respectively, corresponding to 15, 10, 6, 5, 5, 4, 6, and 6 2-DE spots.

Interestingly, many of the proteins involved in central metabolic pathways (e.g., glycolysis, the citric acid cycle, pyruvate metabolism) were identified in both the I-1 and I-2 proteomes. Using the KEGG PATHWAY database (http://www.genome.jp/kegg/pathway.html), we classified more of the I-1 proteins than the I-2 proteins as involved in carbon fixation, glyoxylate and dicarboxylate metabolism, glycolysis/gluconeogenesis, and oxidative phosphorylation (Figure 7). For the two internodes, seven enzymes (15 spots) were classified as carbon fixing, and 13.2% of the I-1 proteins belonged to this category, whereas only 7.7% of those from I-2 did. The difference is related to the number of spots found for the RuBisCo complex, i.e., more were found for I-1 than for I-2. Most of the enzymes of the citrate cycle, pyruvate metabolism, fructose and mannose metabolism, and starch and sucrose metabolism were identified in the two proteomes, and the numbers of proteins found for each pathway were similar, suggesting that the housekeeping pathways are needed for stolon viability. Many enzymes involved in the ubiquitin-proteasome pathway were also identified but were greater in relative number in the I-2 proteome (Figure 7). In plants, regulated protein degradation by the ubiquitin-proteasome system contributes substantially to development by affecting many processes, e.g., embryogenesis, hormone signaling, and senescence, which suggests that the ubiquitin-proteasome system may play a central role in I-2 morphogenesis. The I-1 and I-2 proteomes, which we have described herein, will allow us to analyze and compare proteins in metabolic pathways and may provide new insights into the regulation and expression of various molecular networks.

### Possible PPI networks and adventitious root-formation mechanisms in I-2

Proteins are the main catalysts, structural elements, signaling messengers, and molecular machines of biological tissues [62]. PPIs are extremely important in orchestrating cellular events. Protein interaction networks provide road maps of cellular pathways. Therefore, many methods are used to characterize PPIs, which include physical interactions and functional linkages [63].

**Figure 8:**
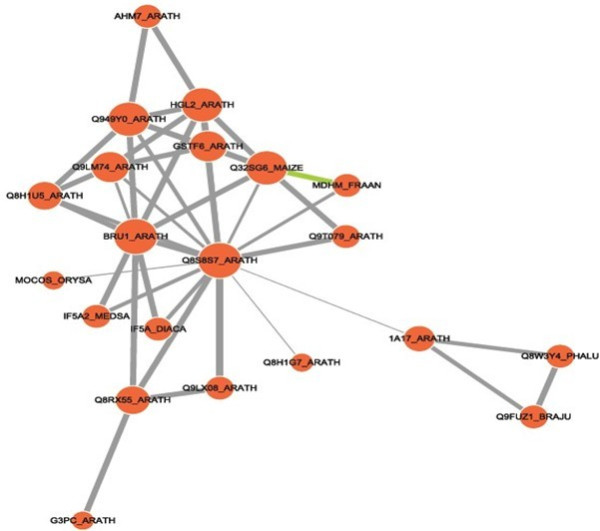
**Possible protein-protein interaction network among I-2 proteins derived using the Cytoprophet module of Cytoscape**. Cytoprophet draws a network of potential interactions with probability scores and GO distances as edge attributes. Proteins are marked with UniProt ID names.

Although we could not use Cytoprophet to delineate a PPI network for I-1 because of limited information, a possible PPI network was found for I-2 (Figure 8). U-box domain-containing protein 34 (PUB34) (Q8S8S7_ARATH) is the central core protein of the signaling network, as it interacts with many other proteins, e.g., alpha, alpha-trehalose-phosphate synthase (TPS4) (Q9T079_ARATH), APC2 (Q8H1U5_ARATH), 1-aminocyclopropane-1-carboxylate synthase 7 (ACS7) (1A17_ ARATH), and EIF5A. APC2 is an E3 ubiquitin ligase that is a component of the SCF family ubiquitin ligases, which catalyze the attachment of ubiquitin to the lysine side chains of securin and mitotic cyclins [64-66]. EIF5A interacts with BRUSHY1 (BRU1_ARATH), which is required for the proper arrangement of cells in the root and shoot apical meristems. Ubiquitin-mediated protein degradation probably affects meristem structural formation by modulating the concentration of cell-cycle regulators and transcription factors [67]. Therefore, the ubiquitin system may be vital during the morphogenesis of clonal plants.

**Figure 9:**
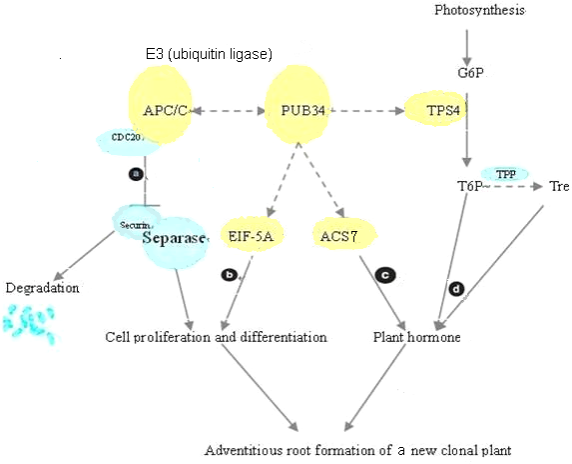
**Model for adventitious root and clonal plant formation in I-2 that incorporates four regulated pathways**. Five identified I-2 proteins were integrated into the model, and the possible PPIs are shown (dashed lines) based on the PPI network in Figure 8. (a) Anaphase-promoting complex (APC/C) is a ubiquitin ligase that plays a key role in the cell cycle. (b) Eukaryotic translation initiation factor 5A (EIF-5A) may interact with PUB34 to regulate cell division. (c) ACS7, when interacting with ubiquitin ligase, plays a central role in ethylene biosynthesis. (d) Important regulatory effects on plant growth and development have been reported for trehalose (Tre) and trehalose 6-phosphate (T6P). (CDC20: cell-division cycle protein 20; G6P: glucose 6-phosphate; TPP: trehalose-6-phosphate phosphatase

Adventitious roots develop from the second node at the end of I-2 before clonal plant formation. We developed a model for adventitious root formation in I-2 based on published data [64-69] and our findings; the model includes four regulated pathways (Figure 9). Regulated protein degradation has repeatedly been identified as a key component of cell-cycle regulation. Securin inhibits a protease called separase, which cleaves cohesins allowing anaphase onset. Activated APC^cdc20 ^targets securin for degradation, which initiates the metaphase-to-anaphase transition [68]. In addition, biochemical and molecular studies have shown that EIF-5A is crucial for plant growth and development as it regulates cell division and cell growth [69]. Continuous cell division, elongation, and differentiation can cause the formation of root primordia, so the APC complex-related and EIF-5A-related biological processes may be two important pathways that regulate the formation of adventitious roots. Moreover, ACS7 catalyzes the conversion of AdoMet into 1-aminocyclopropane-1-carboxylate, a direct precursor of ethylene, whereas ACS7 is ubiquitinated. Ubiquitination probably leads to its subsequent degradation, thus controlling ethylene production. Ethylene can regulate root initiation and emergence. Conversely, as an important catalytic enzyme, TPS4 plays a central role in the complex signaling network that links sugars and hormones with its interacting partner PUB34. Together, the four pathways work synergistically to induce formation of adventitious roots.

## Conclusions

For this research, we compared the proteomes of the I-1 and I-2 internodes of the strawberry stolon to begin to elucidate the how the differences in the proteomes affect the growth and functional characteristics of the two internodes as only the second node, at the end of I-2, tends to form adventitious roots and a clonal plant. In I-1, the majority of the proteins were involved in metabolism, photosynthesis, energy, and transcription. In I-2, relatively more proteins were involved in photosynthesis, carbohydrate metabolism, stress responses, and protein folding and degradation, indicating that these many different processes work synergistically to induce cell differentiation necessary for root and plant formation. Given our findings and those of others, we present a scheme for protein interactions that could be responsible for adventitious root and clonal plant formation in I-2.

## Competing interests

The authors declare that they have no competing interests.

## Authors' contributions

XF designed and carried out experiments, analyzed data, and wrote the manuscript. HM designed the study, interpreted data, and assisted with the writing of the manuscript. DL did most of the MS analysis and assisted with the evaluation of MS data. HY conceived the study and participated in its design. WL designed and analyzed experiments. SR conceived of, designed, and coordinated the study, and assisted with the writing of the manuscript. All authors read and approved the final manuscript.
